# Nutritional analysis and characterization of carbapenemase producing-*Klebsiella pneumoniae* resistant genes associated with bovine mastitis infected cow’s milk

**DOI:** 10.1371/journal.pone.0293477

**Published:** 2023-10-27

**Authors:** Mr. Saddam, Muddasir Khan, Muhsin Jamal, Sadeeq Ur Rahman, Abdul Qadeer, Imad Khan, Mohamed H. Mahmoud, Gaber El-Saber Batiha, Syed Hussain Shah

**Affiliations:** 1 Department of Microbiology, Abdul Wali Khan University, Marden, Pakistan; 2 Centre of Biotechnology and Microbiology, University of Peshawar, Peshawar, Pakistan; 3 College of Veterinary Sciences and Animal Husbandry, Abdul Wali Khan University, Mardan, Pakistan; 4 Shanghai Tenth People’s Hospital, Institute for Infectious Diseases and Vaccine Development, Tongji University School of Medicine, Shanghai, China; 5 Department of Biochemistry, College of Science, King Saud University, Riyadh, Saudi Arabia; 6 Department of Pharmacology, Faculty of Veterinary Medicine, Damanhour University, Damanhour, AlBeheira, Egypt; 7 Department of Health and Biological Sciences, Abasyn University Peshawar, Peshawar, Pakistan; Abadan University of Medical Sciences, ISLAMIC REPUBLIC OF IRAN

## Abstract

The current study was designed to analyze nutritional parameters and to characterize carbapenemase producing-*Klebsiella pneumoniae* isolates from bovine mastitic cow’s milk. Out of 700 milk samples *K*. *pneumoniae* was identified by phenotypic and molecular techniques along with their antibiogram analysis and nutritional analysis was performed using the procedure of Association of Official Analytical Chemists. Carbapenemase-producing *K*. *pneumoniae* was detected by phenotypic CarbaNP test followed by molecular characterization of their associated resistant genes *bla*_*VIM*_, *bla*_*KPC*_, *bla*_*OXA-48*_, *bla*_*NDM*,_ and *bla*_*IMP*_ along with insertion sequence common region 1 (*ISCR1*) and integrons (Int1, Int2, and Int3) genes. Among nutritional parameters, fat content was observed (2.99%) followed by protein (2.78%), lactose (4.32%), and total solid (11.34%), respectively. The prevalence of *K*. *pneumoniae* among bovine mastitis was found 25.71%. Antibiogram analysis revealed that more effective antibiotics was ceftazidime (80%) followed by amikacin (72%), while highly resistant antibiotics was Fusidic acid (100%). Distribution of carbapenemase producer *K*. *pneumoniae* was found 44.4%. Among carbapenem resistant genes *bla*_KPC_ was found 11.25%, *bla*_VIM_ 2.75%, *bla*_NDM_ 17.5%, and *bla*_OXA-48_ 7.5%, while *bla*_IMP_ gene was not detected. Furthermore, distribution of *ISCR1* was found 40%, while integron 1 was found 61.2% followed by integron 2 (20%), and integron 3 (5%). In conclusion, the recent scenario of carbapenemase resistant *K*. *pneumoniae* isolates responsible for mastitis may affect not only the current treatment regime but also possess a serious threat to public health due to its food borne transmission and zoonotic potential.

## Introduction

Milk is crucial for rural development and is also an essential component of a community that is healthy [1]. Milk is made up of proteins, lipids, minerals, carbs, a variety of vitamins, and other specific ingredients scattered in water. Dairy products’ flavor and enjoyment are influenced by the milk’s chemical makeup. Their composition varies by the method of milking, lactation, nutrition, feeding apparatus, habitat, variation of season, and high species diversity [2]. Casein and whey are the two primary kinds of milk protein, both of which are present in varying amounts. Proteins with the casein and whey amino acid profiles have special properties in human vitamins. Casein and whey proteins are categorized as high-quality proteins with quick utilization, high digestion rates, and rapid absorption rates [3].

Even though milk is a nutrient-dense diet, the quantity of nutrients fosters a favorable environment for the development of many microorganisms. The development of dairy products is directly impacted by the microbial makeup of milk [4]. Widespread infectious illness of the mammary glands is called bovine mastitis. They adversely effects on milk productivity, milk quality, pre-term drying-off, culling losses due to hyper mortality, increased veterinary care, therapeutic cost, and livestock welfare aspects as well as various health related issues. Pathogenic bacteria enter into mammary glands by disrupting the physical barriers and cause mastitis [5].

The mastitis in which the quality and quantity of milk productivity is severely affected with non-detectable clinical signs are known as subclinical mastitis. However, clinical mastitis not only affects the productivity but abnormal morphology of mammary glands with other symptoms like changes in appetite and body temperature, etc. [6]. Contagious bacteria that are linked to mastitis quickly infiltrate the mammary glands and are passed from one cow to another by a skin lesion, milking equipment, or flies. *Streptococcus agalactiae* and *Staphylococcus aureus* are the most frequent causes of contagious mastitis [7]. *Escherichia coli* and *Enterobacterales* such as *Klebsiella pneumoniae*, are the primary (90%) causes of mastitis. Among these, *K*. *pneumoniae* is an opportunistic environmental mastitis associated bacterial pathogen prominently reported all over the world [8, 9].

In addition to this, it is important to investigate the recent global rise in the development and spread of antibiotic resistance among gram negative isolates. The huge amount of antibiotics usage in livestock population; one-third of total antibiotic consumption is just used in the treatment of mastitis. The most frequently used antibiotics include tetracycline, streptomycin, cephalosporin and penicillin. Their usage varies between and among the different region of the countries due to variation in health legislation [10]. Several countries, including India [11], Italy [12], Japan [13], and the United Kingdom [14], have documented the existence of extended spectrum beta-lactamase resistance related multidrug resistant (MDR) (ESBL-MDR) *K*. *pneumoniae* isolates. However, the emergence of carbapenemase producing *K*. *pneumoniae* responsible for mastitis has been reported somewhere else [15]. Although, limited data with diverse genetic information of heterogeneous nature among bovine mastitis associated nutritional analysis and *K*. *pneumoniae* strains remain the topic of debate. The current study was designed in order to investigate the nutritional analysis, prevalence of carbapenemase-producing *K*. *pneumoniae*, and molecular identification of their resistance genes linked to bovine mastitis in dairy farms in Peshawar, province of Pakistan.

## Materials and methods

### Ethical approval

The study was conducted in accordance with national and institutional regulations after receiving ethical permission from the Ethical Review Board of Abdul Wali Khan University, Mardan, Pakistan.

### Study location and sample collection

The current investigation was carried out in District Peshawar (34.0151°N, 71.5249°E), the capital of Pakistan’s Khyber Pakhtunkhwa Province, from September 2018 to August 2019. In the fall and summer months, total 700 composite clinical mastitic cows were sampled. The distribution of the cows was based on their demographic traits, such as farm type, recurrence status, medication, and lactation stage. In a 50 ml sterile container, 10 ml of milk samples were taken from each animal’s mammary gland quarter. For further processing, the milk samples were transferred aseptically at 4°C to the microbiology laboratory at the college of veterinary sciences at Abdul Wali Khan University.

### Nutritional analysis

The Association of Official Analytical Chemists’ protocol was used to analyze the nutritional content of milk. At the Veterinary Research Institute Peshawar (VRI), the experiment was conducted in the livestock research and development section. Ashes were measured after samples were incinerated at 550°C for 6 hours after being dried at 105°C for 24 hours to remove moisture. With the use of this technique, many other elements, including crude fat, dry matter, crude protein, and fibers were measured [16].

### Isolation and characterization of *K*. *pneumoniae* isolates

The milk sample were inoculated in Simon Citrate Inositol agar (SCHI) plates and were incubated for 24–48 h at 37°C.Glycerol stabs were used for storage of grown colony at −80°C for further process which can be easily recovered by inoculation on agar plates, followed by incubating for 24 h at 37°C. *K*. *pneumoniae* were characterized and identified by morphological and biochemical technique followed by molecular confirmation through *16sRNA* gene by using a set of primers (Table 1) and *K*. *pneumoniae* 10271 was used as bacterial control species [17].

**Table 1:** List of studied carbapenem resistant *K*. *pneumoniae* targeted genes and primers.

Gene	Sequence	Temperature	Base pair (Bp) Size	Reference
*16sRNA*	F (ATT TGA AGA GGT TGC AAA CGA T)	58°C	260bp	[21]
	R (CCGAAG ATG TTT CAC TTC TGA TT)			
*VIM*	F (GATGGTGTTTGGTCGCATA)	55°C	385bp	[23]
	R (GAATGCGCAGCACCAGGATA)			
*NDM*	F (GGTTTGGCGATCTGGTTTTC)	55°C	621bp	[23]
	R (CGGAATGGCTCATCACGATC)			
*KPC*	F (CGTCTAGTTCTGCTGTCTTG)	55°C	798bp	[23]
	R (CTTGTCATCCTTGTTAGGCG)			
*OXA-48*	F (GCGTGGTTAAGGATGAACAC)	55°C	438bp	[23]
	R (CATCAAGTTCAACCCAACCG)			
*IMP*	F (GGAATAGAGTGGCTTAACTCTC)	55°C	232bp	[23]
	R (GGTTTAACAAAACAACCACC)			
*Int1*	F (CCT CCC GCA CGA TGA TC)	54°C	280bp	[24]
	R (TCC ACG CAT CGT CAG GC)			
*Int2*	F (AAA TCT TTA ACC CGC AAA CGC)	54°C	439bp	[24]
	R (ATG TCT AAC AGT CCA TTT TTA AAT TCT A)			
*Int3*	F (AGT GGG TGG CGA ATG AGT G)	54°C	599bp	[24]
	R (TGT TCT TGT ATC GGC AGG TG)			
*ISCR1*	F (CGC CCA CTC AAA CAA ACG)	55°C	569bp	[25]
	R (GAG GCT TTG GTG TAA CCG)			

### Antimicrobial susceptibility of carbapenem-resistant isolates

CarbaNP test were used for the identification of Carbapenem-resistant isolates according to guidelines of Clinical and Laboratory Standard Institute [18, 19] where *K*. *pneumoniae* 9014 was used as a reference strain [20]. Phenotypic characterization and screening was determined using a Kirby-Bauer disk diffusion assay according to CLSI, 2017. A total of 14 antibiotics including amoxicillin + clavulanic acid, Amoxicillin, Ceftazidime, Fusidic acid, Chloramphenicol, Ciprofloxacin, levofloxacin, sulphametizine, Cefepime, Amikacin, Gentamicin, Tetracycline and Imipenem were used for antibiogram analysis of the entire carbapenem resistant *K*. *pneumoniae* isolates. The hyper-virulent *K*. *pneumoniae* phenotype was analyzed by the string test as per the recommendations of Rodriguez-Medina and co-workers [21].

### Molecular identification of carbapenem resistance genes

Overall *K*. *pneumoniae* isolates that were phenotypically confirmed as carbapenem resistant were examined for the presence of class A carbapenemases genes, which include; *bla*_KPC-type_, metallo-β-lactamases genes including *bla*_VIM-type_, *bla*_IMP-type_, *bla*_NDM-type_ genes and carbapenemase associated integrons (Int1, Int2, and Int3) with insertion sequence common region 1 (ISCR1) using specific primers (Table 1). The Polymerase chain reaction (PCR) was optimized and performed followed by previously described method for each family [22].

### Statistical analysis

The obtained results were analyzed and organized using Microsoft Excel and Word. The descriptive statistics of obtained values were analyzed using Statistical Packages of Social Sciences (SPSS) 23.0 version and Graph pad prism 8.0.

## Results

### Nutritional analysis

**Fig 1:**
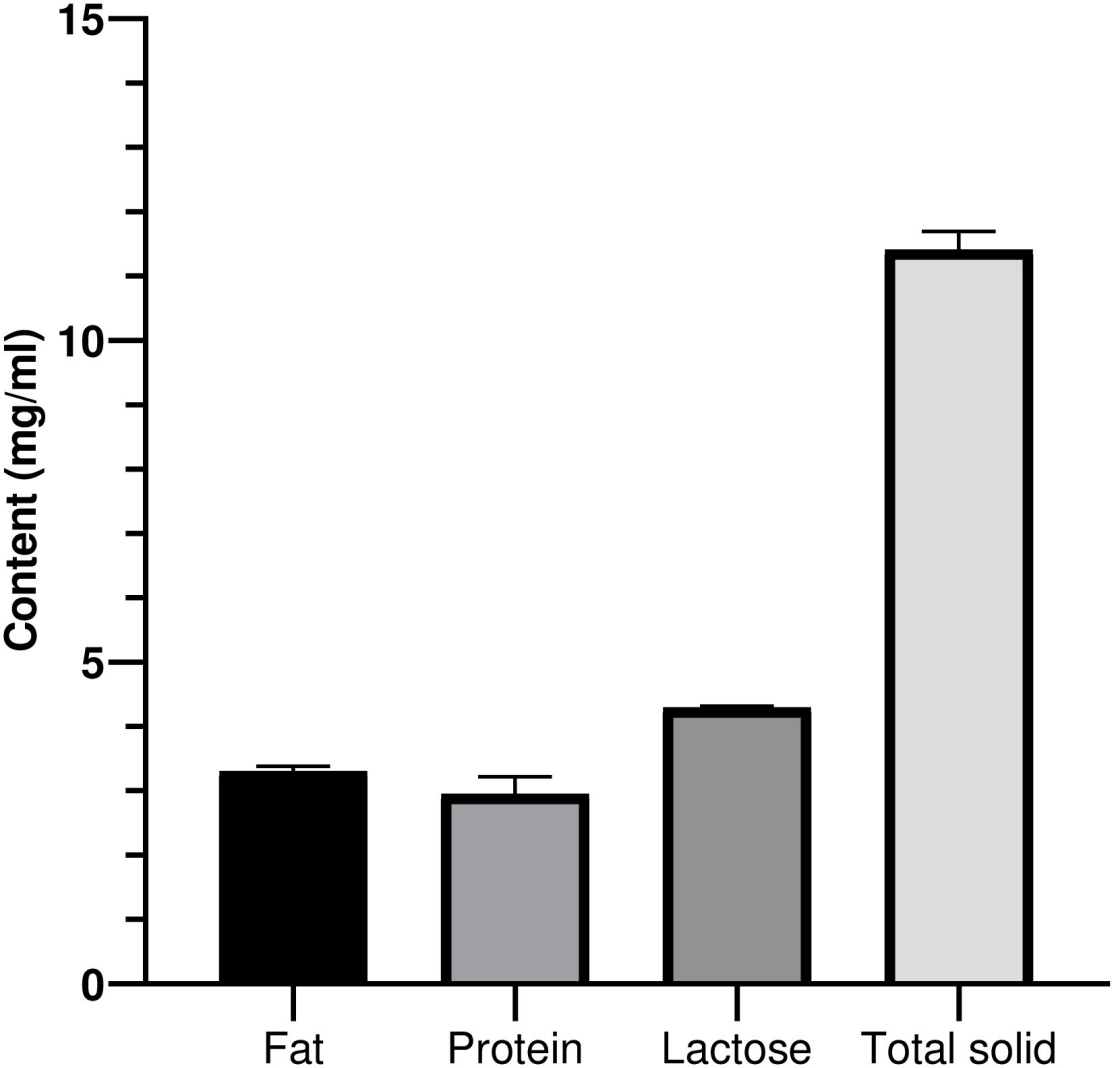
Nutritional analysis of milk samples collected from bovine mastitis infected cows.

In the present study, among the total 700 milk samples collected from bovine mastitis infected cows, the fat content was found (2.99%) with Mean ± SD (3.36 ± 0.11). The protein content was found (2.78%) with Mean ± SD (2.76 ± 0.04). The lactose content was found (4.32%) with Mean ± SD (4.31 ± 0.10). The total solid content was found (11.34%) with Mean ± SD (11.61 ± 0.27) (Fig 1).

### Prevalence of *K*. *pneumoniae* and their antibiogram

**Fig 2:**
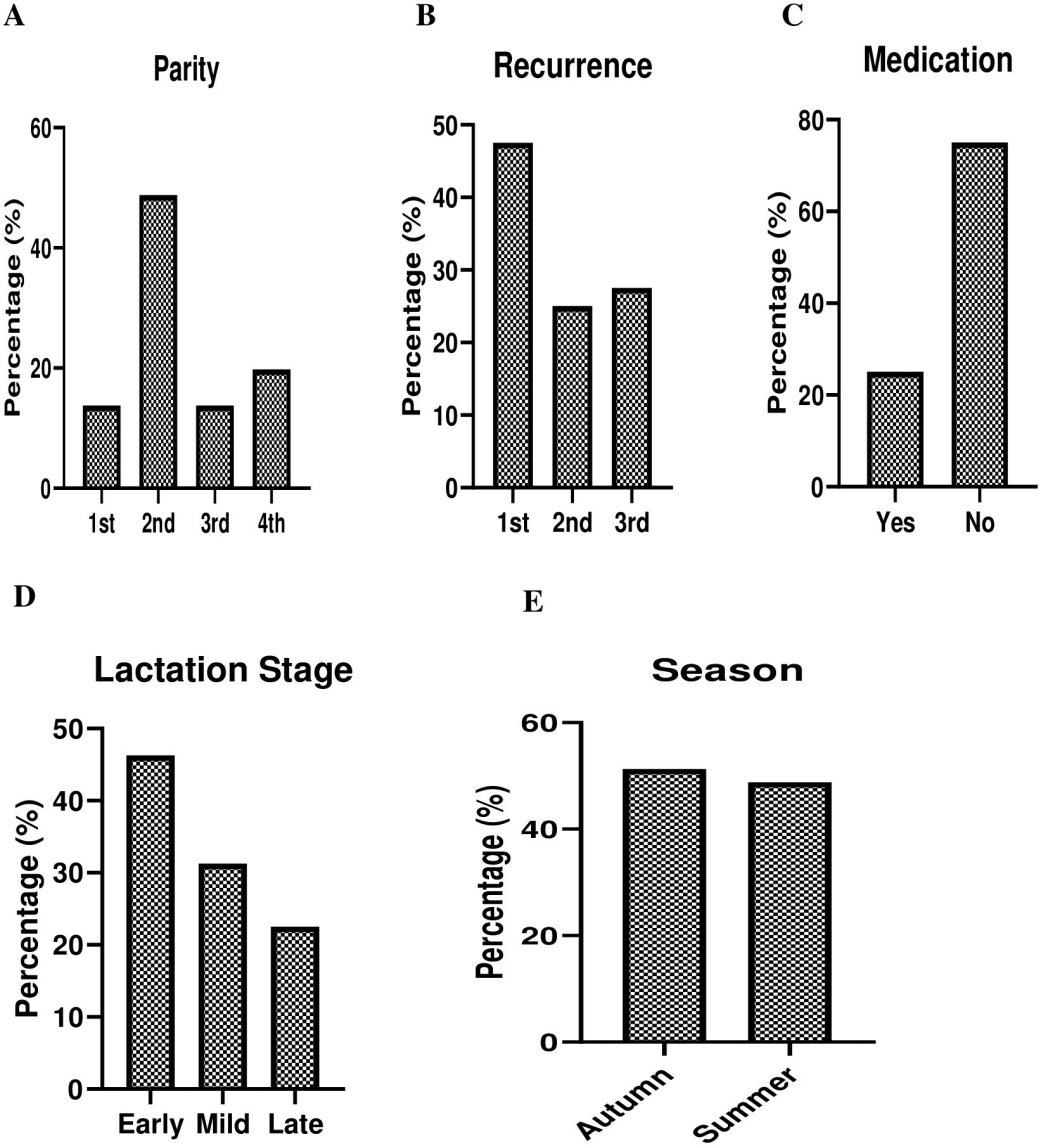
Carbapenem resistant *K*. *pneumoniae* demographical distributions; a) Parity, b) Recurrence status, c) Medication, d) Lactation stage, and e) Season.

**Fig 3:**
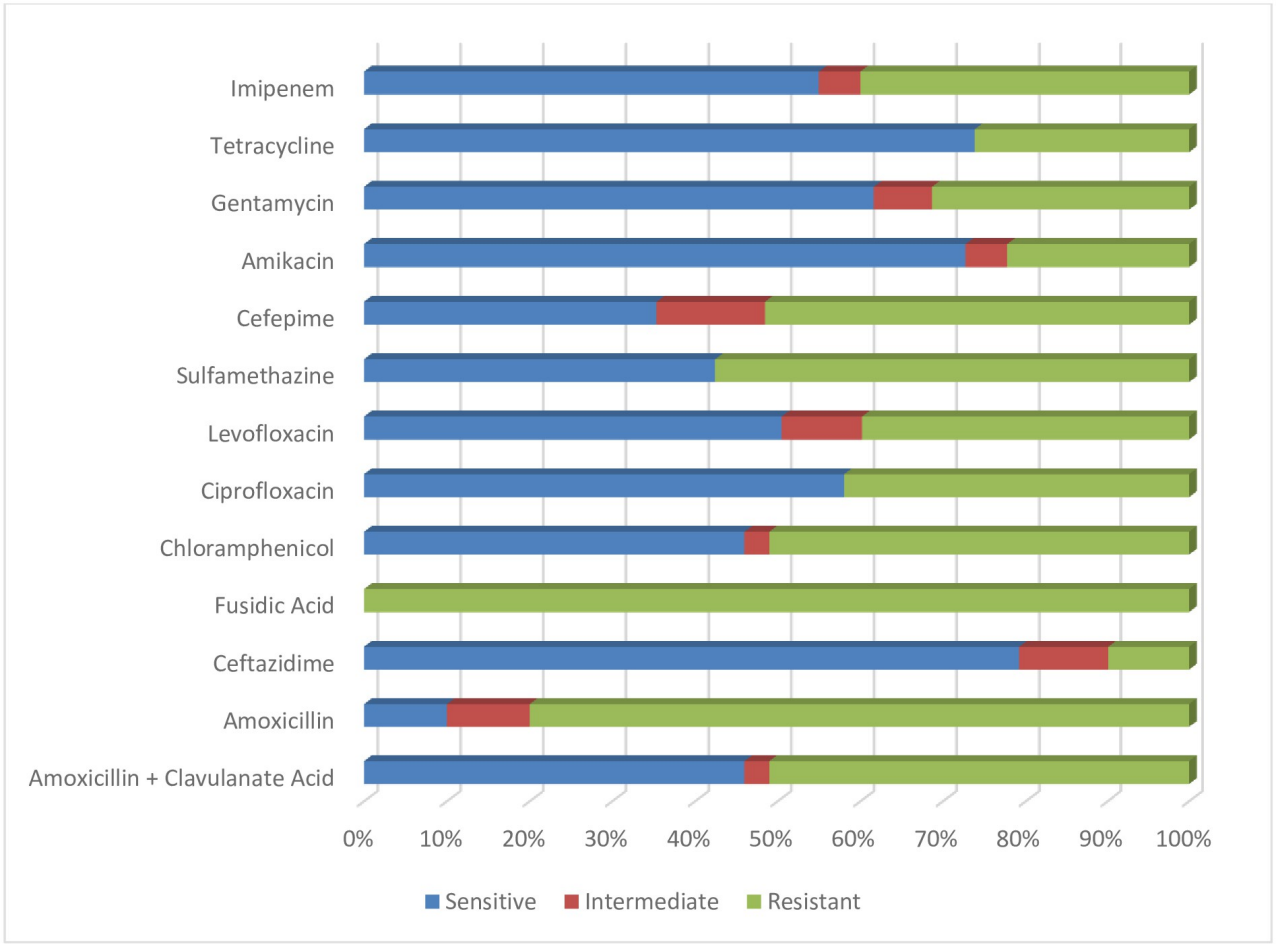
Antibiogram analysis of *K*. *pneumoniae* isolates from bovine mastitis cows’ samples (S1 Table).

Among the total 700 milk samples, *K*. *pneumoniae* was found in 180 / 700 (25.7%). These isolates were confirmed by *16sRNA* gene detection. Furthermore, among these isolates carbapenem resistant *K*. *pneumoniae* was found 80 / 180 (44.44%) milk samples. These resistant cows’ parity, recurrence status, medication, lactation stage, and season were presented in Fig 2. All 80 / 180 (44.44%) isolates were found multi drug resistant (MDR) by their antibiogram analysis. Briefly these isolates were found highly sensitive to Ceftazidime (80%) followed by amikacin (72%) antibiotics while found highly resistant to Fusidic acid (100%) antibiotics (Fig 3).

### Carbapenem resistance genes

**Fig 4:**
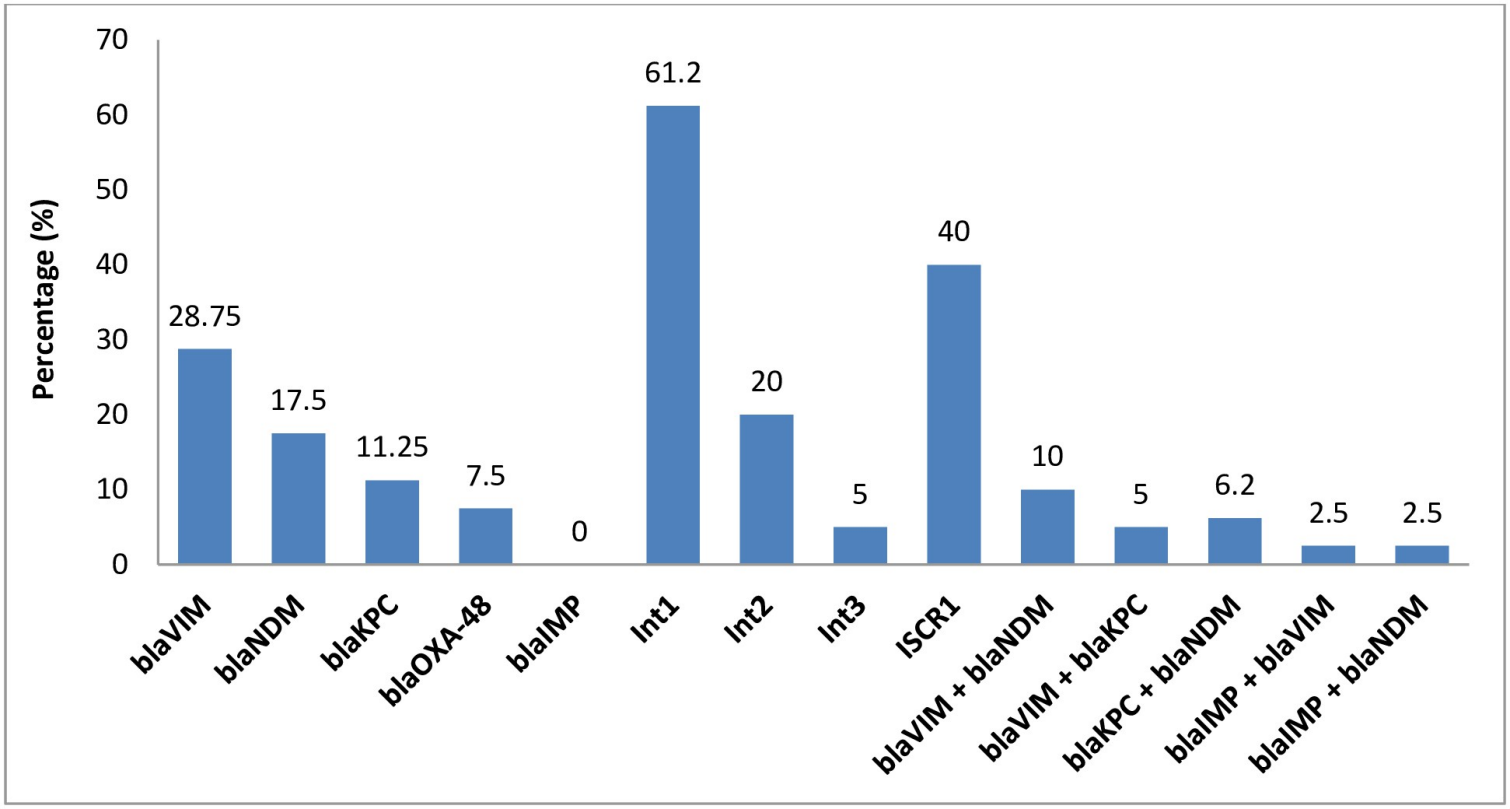
Distribution of carbapenem resistant genes among *K*. *pneumoniae* isolates from bovine mastitis.

The carbapenem resistant genes among these isolates (S1 Fig) were distributed as *bla*_KPC_ (11.25%), *bla*_VIM_ (28.75%), *bla*_NDM_ (17.5%), and *bla*_OXA-48_ (7.5%), while *bla*_IMP_ gene was not detected in any isolates; furthermore the co-occurrence of these genes was presented in Fig 4. The distribution of *ISCR1* was found 40%, the other detected integrons distribution was presented in Fig 4. Further area wise distributions of these genes were presented in Table 2.

**Table 2:** Area-wise diversity of carbapenemase producing-*K*. *pneumoniae* resistance genes in district Peshawar, Pakistan.

Location	Resistance Antibiotics	Carbapanemase genes				Co-occurrence				
		KPC	VIM	OXA	NDM	VIM+NDM	KPC+ VIM	NDM+KPC	IMP+VIM	IMP+NDM
Landi arbab	CAZ, AML, AK, IMP, C, FD, SXT, FEP, TE	+			+			+		
Pishtakhara Bala	IMP, CAZ, FD, AML, AK, SXT, TE, FEP, C		+							+
Sarband	FEP, C, TE, SXT, AK, FD, AML, CAZ, IMP		+		+	+				
Spina warai	CAZ, AML, IMP, FD, TE, SXT, FEP, C, AK			+					+	
Sangu	TE, IMP, AK, CAZ, FEP, SXT, FD, C, AML	+								
Masho khel	IMP, CAZ, AK, C, SXT, AML, FD, TE, FEP		+		+	+				
Bazid khel	SXT, FEP, TE, AML, CAZ, C, AK, IMP, FD		+							+
Sheikh Muhammadi	FD, C, TE, IMP, SXT, CAZ. AML, AK, FEP			+						
Gharib abad	CAZ, AML, AK, IMP, C, FD, SXT, FEP, TE		+		+			+		
Urmar	IMP, CAZ, FD, AML, AK, SXT, TE, FEP, C,	+	+		+		+	+		
Bara	FEP, C, TE, SXT, AK, FD, AML, CAZ, IMP		+							
Mattani	CAZ, AML, IMP, FD, TE, SXT, FEP, C, AK		+		+					
Bara gate	TE, IMP, AK, CAZ, FEP, SXT, FD, C, AML		+							
Gulbahar	IMP, CAZ, AK, C, SXT, AML, FD, TE, FEP		+		+	+				
Custom Chowk	SXT, FEP, TE, AML, CAZ, C, AK, IMP, FD	+	+				+			
Nouthia	FD, C, TE, IMP, SXT, CAZ. AML, AK, FEP			+	+	+				
Hazar khwani	FEP, C, TE, SXT, AK, FD, AML, CAZ, IMP		+						+	
Duran pur	CAZ, AML, IMP, FD, TE, SXT, FEP, C, AK		+		+					
Chamkani	TE, IMP, AK, CAZ, FEP, SXT, FD, C, AML		+	+						
Phandu	IMP, CAZ, AK, C, SXT, AML, FD, TE, FEP	+	+		+			+		
Khazana Chowk	SXT, FEP, TE, AML, CAZ, C, AK, IMP, FD	+	+				+			
Taj Abad	FD, C, TE, IMP, SXT, CAZ. AML, AK, FEP		+		+	+				+
Bakhshi pul	CAZ, AML, AK, IMP, C, FD, SXT, FEP, TE	+	+							
Javad town	IMP, CAZ, FD, AML, AK, SXT, TE, FEP, C			+	+	+				
Jameel Chowk	FEP, C, TE, SXT, AK, FD, AML, CAZ, IMP	+		+						
Sukanu	CAZ, AML, IMP, FD, TE, SXT, FEP, C, AK				+					
Faqir Abad	SXT, FEP, TE, AML, CAZ, C, AK, IMP, FD		+		+	+				
Khazana	FD, C, TE, IMP, SXT, CAZ. AML, AK, FEP	+		+		+		+		
Gul Badar	TE, IMP, AK, CAZ, FEP, SXT, FD, C, AML	+								
Bakshu	IMP, CAZ, AK, C, SXT, AML, FD, TE, FEP		+							
Shekhan	SXT, FEP, TE, AML, CAZ, C, AK, IMP, FD				+					
Kababyan	FD, C, TE, IMP, SXT, CAZ. AML, AK, FEP		+							
Shahkas	CAZ, AML, AK, IMP, C, FD, SXT, FEP, TE				+					
Shaheeb Town	IMP, CAZ, FD, AML, AK, SXT, TE, FEP, C		+							
Phase 3 Chowk	FEP, C, TE, SXT, AK, FD, AML, CAZ, IMP		+							
Kakshala	CAZ, AML, IMP, FD, TE, SXT, FEP, C, AK				+					

## Discussion

Milk comes from a variety of animals, including goats, cows, buffalo, sheep, and even people. It is a nutrient-dense diet. However, the quantity of macro and micronutrients as well as vitamins and minerals in different types of milk provides an ideal environment for the development of many bacteria. Nutrients can either be readily available for all bacteria or need specific populations to break down main components to release nutrients and metabolites that can be utilized by other microbes. The growth and production of dairy products is directly affected by the microbial makeup of milk [4]. Previously, Antanaitis et al. [26] reported the low level of lactose in mastitis infected milk. Similarly in agreement, we found the fat content (2.99%), protein content (2.78%), lactose content (4.32%), and total solid content (11.34%).

Bovine mastitis is a serious multi-pathogenic inflammatory infection of the mammary tissue in dairy cows, is to blame for the widespread use of antibiotics for treatment as well as for maintaining animal health and the livestock industry’s financial stability [27–30]. Massive antibiotic overuse without a prescription causes therapeutic failure in dairy cows against mastitis-causing bacteria such *K*. *pneumoniae* [31]. *K*. *pneumoniae* is a significant opportunistic pathogen that causes pneumonia, bacteremia, septicemia, and mastitis in a variety of animal and human worldwide [32–34]. Unchecked and excessive use of antibiotics for growth promotion and therapeutic purposes in animal’s results in the emergence of virulence genes associated with multidrug resistance, such as carbapenem, in isolates of *K*. *pneumoniae* linked to mastitis.

The current study observed high prevalence of *K*. *pneumoniae* (25.7%) which is similar to previous observation of lower prevalence of sub-clinical mastitis among livestock population by Younas et al. [35]. However, opposite results have been reported by Hussain et al. [36], Ali et al. [37] from Pakistan. Likewise results of higher prevalence of sub-clinical mastitis were observed from Sudan and Ethopia by Nigo et al. [38], Amin et al. [39]. The similarities and deviations in prevalence of mastitis among various studies from different geographical locations might be due to the involvement of complex etiological agents, diagnostic protocols and other factors like environmental hygiene, dairy farm management, herd size and quarter levels [36].

The rapid emergence of carbapenem-resistant bacterial isolates in livestock, food-producing animals, and wildlife is currently a significant public health concern that further muddies the picture of antibiotic resistance worldwide [15]. It is generally known that *K*. *pneumoniae* has become resistant to carbapenems [40]. Significant increases in disease and death are attributed to rising antimicrobial drug resistance, especially carbapenem-resistant *K*. *pneumoniae* (CRKP) [41]. For infections brought on by CRKP, few antimicrobial therapeutic alternatives are available [42]. Interestingly, in the current investigation of carbapenem resistant *K*. *pneumoniae* isolates linked with mastitis, Ceftazidime and amikacin had the highest sensitivity levels. Amikacin was shown to be effective against all *K*. *pneumoniae* isolates [43]. Amikacin was also found to be effective against bacteria isolated from the urine of individuals with infected urinary tracts [44]. It is notable that there was little cross-resistance to tetracycline among CRKP and that *K*. *pneumonia* resistance rate remained constant over the research period [45]. Because they have better tissue penetration, antibacterial activity, and a lower propensity to acquire antimicrobial drug resistance than their older counterparts, later-generation tetracyclines may be helpful in the treatment of CRKP-related illnesses [46]. Tigecycline is a glycylcycline antibacterial drug that has been used to treat CRKP-related infections and has been seen there the most frequently effective against *K*. *pneumoniae* that produces carbapenemase [47, 48]. For infections brought on by carbapenemase-producing *K*. *pneumoniae* (KPC-Kp), Ceftazidime/avibactam is a crucial therapy choice [49]. The urgency to strengthen preventative efforts and treatment approaches is increased by the rising antimicrobial drug resistance to *K*. *pneumoniae* in our study, a parallel lack of innovative antimicrobial agent development [45], and the dearth of effective therapeutic choices for treating CRKP-related illnesses [48]. Several countries highlight the potential contribution of animals raised for food as a source of resistant microorganisms. Pigs and hens are the animals that have been investigated the most, and carbapenem resistance in Enterobacteriaceae and non-fermenting bacteria has been seen there the most frequently [50, 51]. The different *K*. *pneumoniae* isolates that are resistant to carbapenems generate either class A (KPC), class B (IMP, VIM, and NDM), or class D (OXA-48) serine carbapenemases [52, 53]. According to recent findings, the presence of both blaKPC (class-A-MBL) and blaIMP, blaVIM, and blaNDM (class-B-MBLs) in *K*. *pneumoniae* isolates from cows with mastitis poses a severe concern to human health since milk can spread a number of bacterial infections. Highlight the potential contribution of animals raised for food as a source of resistant microorganisms [14]. There have been several reports of *K*. *pneumoniae* generating the blaKPC-2 gene in animals and the environment since the initial report of *K*. *pneumoniae* producing KPC in 1996 [54–56]. Similar to the previous study, *K*. *pneumoniae* ST258 from the pandemic clone was found in waste water treatment plants in Austria [58] and a hospital effluent and wastewater treatment facility in Brazil [57]. Previously isolated from human patients in a number of nations, including the UK [53], Singapore [54], Algeria [55], Japan [56], Denmark [57], The Netherlands [58], Spain [59], the USA [60], Australia [61], and China [62], *K*. *pneumoniae* having blaNDM-5 genes was found in milk and feces of dairy cows with mastitis in Jiangsu Province, China [32]. On the other hand, blaVIM-resistant isolates of *K*. *pneumoniae* have also been found in neonates admitted to ICUs in Naples, Italy [63], *Aeromonas caviae* isolates from clinical surveillance cultures in Israeli hospitals [64], and *A*. *caviae* isolates from blood cultures of 1-day-old newborns in Florence, Italy [65]. These isolates are VIM-producing isolates with a worldwide geographic distribution and are isolated in several Enterobacteriaceae species [66]. These findings highlight the need of improved farm management to stop the spread of *K*. *pneumoniae* that produces carbapenemase. Access to precise diagnostic methods for ongoing monitoring, early diagnosis of mastitis bacteria, and rapid treatment of the condition is advantageous for global public health [15]. Furthermore, rapid action needed to implement preventative strategies and identification of the causative agent along with their resistance profiles. Additionally, characterizing bacterial strains from sick animals makes it easier to pinpoint the origins of infection and determine if herd infections are polyclonal or clonal in origin. This information enables understanding the mode of transmission within herds [67–70]. To the best of our understanding, this work is the first to document the discovery of *K*. *pneumoniae* isolates that produce blaVIM in samples originating from cases of bovine mastitis, most likely as a result of animal feces contaminating the water or food supply.

## Conclusions

It is concluded from the current scenario of high prevalence of carbapenem resistant *K*. *pneumoniae* isolates of mastitis origin effect not only the current treatment regime but also possess a thoughtful threat to public health due to its food born transmission and zoonotic potential. Furthermore, the nutritional analysis can also be used for mastitis early detection. Moreover, the empirical therapeutic approach should be minimized, antibiogram analysis should be properly followed, and recommended therapeutic drugs in proper dosage form should be used against the isolated bacterial strain.

## Supporting information

S1 Fig Electrophoretogram of amplified carbapenem resistant genes from *K*. *pneumoniae* isolates, (A) 16sRNA, (B) *Int1*, *Int2*, *Int3*, *ISCR1*, (C) KPC, VIM, NDM, OXA-48. (DOCX)

S1 Table Antibiogram analysis of carbapenemase producing *K*. *Pneumoniae* isolated from milk of cows. (DOCX)
