# Correction: Nutritional analysis and characterization of carbapenemase producing-*Klebsiella pneumoniae* resistant genes associated with bovine mastitis infected cow’s milk

**DOI:** 10.1371/journal.pone.0297123

**Published:** 2024-01-10

**Authors:** Mr. Saddam, Muddasir Khan, Muhsin Jamal, Sadeeq Ur Rahman, Abdul Qadeer, Imad Khan, Mohamed H. Mahmoud, Gaber El-Saber Batiha, Syed Hussain Shah

There are errors in the Funding section. The correct Funding statement is: The authors extend their appreciation to the Researchers Supporting Project number (RSP-2023-R406), King Saud University, Riyadh, Saudi Arabia, for funding this research. We are also thankful to the Higher Education Commission of Pakistan for funding the research experimental work under the NRPU project (Sr. Number: 8633). The funders had no role in study design, data collection and analysis, decision to publish, or preparation of the manuscript.
