# Supplementary figures and images for: Nutritional analysis and characterization of carbapenemase producing-Klebsiella pneumoniae resistant genes associated with bovine mastitis infected cow’s milk
Source: PLoS One. 2023 Oct 27;18(10):e0293477. doi: 10.1371/journal.pone.0293477 (PMC10610456; doi:10.1371/journal.pone.0293477)

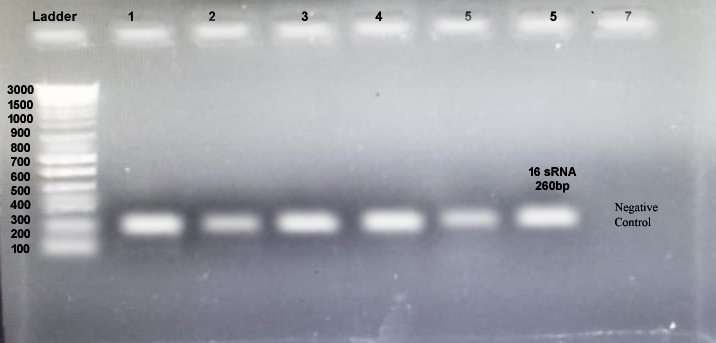


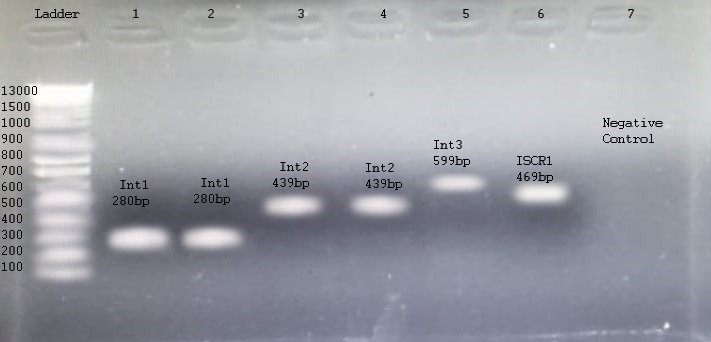


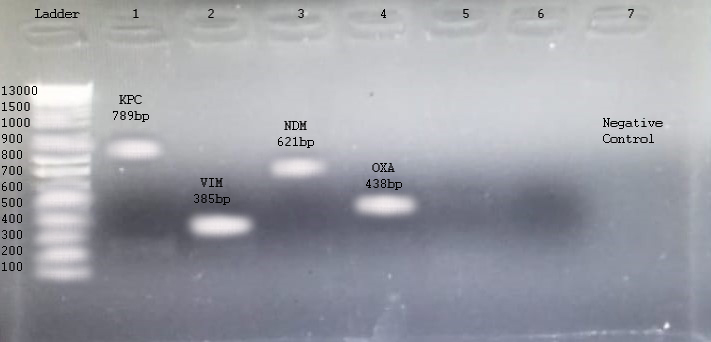

Supplement: S1 Fig — (DOCX) [file pone.0293477.s001.docx]
