# Supplementary material for: Nutritional analysis and characterization of carbapenemase producing-Klebsiella pneumoniae resistant genes associated with bovine mastitis infected cow’s milk
Source: PLoS One. 2023 Oct 27;18(10):e0293477. doi: 10.1371/journal.pone.0293477 (PMC10610456; doi:10.1371/journal.pone.0293477)
Supplement: S1 Table — (DOCX) [file pone.0293477.s002.docx]

| Antibiotic Disc | Abbreviation | Sensitive | Resistant | Intermediate |
| --- | --- | --- | --- | --- |
| Amoxicillin + Clavulanate Acid | AMC | 37/80(46%) | 41/80(51%) | 2/80(3%) |
| Amoxicillin | AML | 8/80(10%) | 64/80(80%) | 8/80(10%) |
| Ceftazidime | CAZ | 64/80(80%) | 8/80(10%) | 9/80(11%) |
| Fusidic Acid | FD | 0/80(0%) | 80/80(100%) | 0/80(0%) |
| Chloramphenicol | C | 37/80(46%) | 41/80(51%) | 2/80(3%) |
| Ciprofloxacin | CIP | 52/80(65%) | 38/80(47%) | 0/80(0%) |
| Levofloxacin | LEV | 42/80(52%) | 30/80(41%) | 8/80(10%) |
| Sulfamethazine | SXT | 34/80(42%) | 46/80(57%) | 0/80(0%) |
| Cefepime | FEP | 28/80(35%) | 41/80(51%) | 11/80(13%) |
| Amikacin | AK | 58/80(72%) | 18/80(22%) | 4/80(5%) |
| Gentamycin | CN | 49/80(61%) | 25/80(31%) | 6/80(7%) |
| Tetracycline | TE | 52/80(65%) | 24/80(23%) | 4/80(5%) |
| Imipenem | IMP | 44/80(55%) | 32/80(40%) | 4/80(5%) |
